# Impact of small residual setup errors after image guidance on heart dose and survival in non-small cell lung cancer treated with curative-intent radiotherapy

**DOI:** 10.1016/j.radonc.2020.04.008

**Published:** 2020-11

**Authors:** Corinne Johnson-Hart, Gareth Price, Alan McWilliam, Andrew Green, Corinne Faivre-Finn, Marcel van Herk

**Affiliations:** a Manchester Cancer Research Centre, Division of Cancer Sciences, School of Medical Sciences, Faculty of Biology, Medicine and Health, University of Manchester, Manchester Academic Health Science Centre, UK; b University of Manchester, Manchester Academic Health Science Centre, The Christie NHS Foundation Trust, UK

**Keywords:** NSCLC, Radiotherapy, Heart dose, Image-based data-mining, Residual setup errors

## Abstract

**Background and purpose:**

A recent study of NSCLC patients showed small residual setup errors (shifts) in the direction of the heart following image-guidance were significantly related to overall survival. This study of the dosimetric effects of these residual shifts investigates the hypothesis that observed survival differences were related to a change in heart dose.

**Materials and methods:**

Accumulated doses including shifts for each fraction were determined for 475 NSCLC patients. Planning CTs and corresponding dose distributions were deformed to a reference. Image-based data-mining techniques were then applied to the difference between the planned and accumulated dose (Δdose) to determine where Δdose relates to 1-year survival. The significance of Δdose in the identified region was assessed using multivariable Cox analysis. The cohort was then split into octiles, based upon planned dose to the region, and multivariable Cox analysis performed for each sub-cohort to explore the dose response relationship. The identified dose threshold for damage was then tested in an independent validation cohort of 1482 NSCLC patients from the same institution.

**Results:**

Permutation testing identified a small region in the heart base where Δdose significantly correlated with 1-year survival. Δdose in this region showed no correlation with common clinical variables, and was significant in multivariable Cox regression (*p* < 0.001, hazard ratio 1.221/Gy), with increasing change in dose from plan resulting in greater risk of death. Octile analysis revealed Δdose to be significant only in the 7th octile, planning dose 16.2–23.4 Gy, suggesting a steep dose–effect relation for heart damage in this range. Taking 16.2 Gy as a conservative threshold dose, this result was successfully validated, with a significant difference being seen between patients with a region dose above or below 16.2 Gy.

**Conclusions:**

This study suggests the relation between residual set-up errors and survival is explained by changes in cardiac dose, and identifies an area at the heart base where dose is correlated with survival. Our results suggest the dose threshold for cardiac damage is between 16.2 and 23.4 Gy in the base of the heart, which was validated in an independent cohort. However, the dose effect in other regions of the heart should also be investigated.

## Highlights

• Residual errors following treatment setup result in a change in dose from planned.
• The change in dose in a region in the heart is related to overall survival.
• No relationship between the change in dose and other clinical variables was found.
• Increased heart dose explains the relationship between residual errors and survival.

Radiotherapy plays an important role in the treatment of lung cancer. Radiotherapy treatments are planned on Computed Tomography (CT) images, acquired with the patient in the treatment position. For accurate delivery, the patient position must be replicated at each fraction, as any changes will result in differences in the delivered dose distribution compared to that planned.

Image guided radiotherapy (IGRT) has been developed over the last two decades to aid patient positioning. Numerous studies have demonstrated its advantages [1], [2], [3], reporting superior conformance to the plan. As such, IGRT is now commonly used for the correction of patient setup errors [4], [5]. For practical reasons, IGRT is often not applied for every treatment fraction, but rather in an off-line fashion i.e. by using measurements from previous fractions to determine how the patient should subsequently be set up [4], [6]. Furthermore, action thresholds are often used, whereby only errors above a pre-defined magnitude are corrected. With such protocols, there will be small residual errors.

A recent study by our group [7] looked at the effect of residual setup errors after IGRT on survival. For a cohort of 780 NSCLC patients no correlations of the residual errors with clinical variables were found, yet the errors were significantly associated with overall survival. Specifically, patients with residual shifts that move the heart towards the high dose region were found to have significantly worse survival compared to patients with residual shifts that move the heart away. It was assumed that the observed survival difference was related to changes in heart dose, which is in line with the results of other studies that found early mortality to correlate with dose in specific heart regions [8], [9], [10], [11].

The aim of this study is to investigate the hypothesis that the observed relationship between residual setup errors and overall survival is due to changes in delivered dose. Using image-based data-mining [12] we aim to identify the anatomical location where the change in dose, due to residual setup errors, correlates with worse survival and investigate the dose threshold for damage.

## Methods

From the original cohort of 780 NSCLC patients treated at a single institution, described by Johnson-Hart et al. [7], a subset of 546 NSCLC patients for whom the planning CT scan, dose and radiotherapy planning structures were available for analysis were selected (patient cohort details described in Supplementary Materials 1). We limited the cohort to the most common radiotherapy regimen (55 Gy in 20 fractions) to remove potential interactions between prescription and baseline prognosis. Full details of the treatment imaging protocol and method to estimate the residual shifts can be found in the original article [7]. Briefly, 3D-CBCT images were acquired prior to treatment delivery at the first 3 fractions and weekly thereafter. These images were rigidly registered to the planning scan based on bony-anatomy to derive the appropriate couch shift. If any of the required shifts were greater than the 5 mm action threshold, then an online correction was performed. Residual setup errors were determined by retrospectively applying the action threshold to the recorded image matches. The vector shift towards the heart was then calculated by determining the difference in the distance between the centre of mass of the target and the heart with and without the residual setup errors applied. For each patient, this vector shift was summarised over the course of their treatment.

Full evaluation of the delivered dose requires many thousands of calculations. We therefore assumed ‘shift invariance’ and estimated the dose at each fraction by shifting the dose distributions relative to the patient, which can be considered as a zeroth order approximation of the true delivered dose. The final accumulated dose distribution is found by summing the daily contributions. The validity of this assumption was tested via comparison with full dose recalculations (using Raystation 6R, Raysearch inc, Stockholm) for a subset of 13 patients, selected to include a range of clinical parameters (e.g. comorbidities, tumour size and position) and representative residual setup errors as seen in the full cohort (Supplementary Materials 2).

For each patient we subtracted the accumulated dose from the planned dose at each voxel to determine a Δdose distribution, in which positive values indicate a higher delivered dose than planned. We then analysed the mean difference in Δdose between patients that were alive or not at 1 year. Δdose was studied as: (i) it is our hypothesized driver of the difference in survival with different residual setup errors, and (ii) its value is independent of other treatment variables (Fig. 2), and thus is not confounded, unlike planned dose which is highly confounded (by e.g. tumour size, location and nearby healthy anatomy). As a result it provides a cleaner signal, which is more sensitive for the detection of smaller effects. Regions of dose associated with patient outcome were determined using the image-based data-mining approach described by Chen et al. [12] and McWilliam et al. [13]. Briefly, patient CTs were non-rigidly registered to a reference patient using NiftyReg [14]. Patients were then grouped based upon survival status at one year and mean Δdose distributions for each group calculated. The difference between the mean Δdose distributions of the groups is subsequently calculated and significance assessed using permutation testing [12]. We used 1000 permutations to test the null hypothesis that there was no difference between the average Δdose distributions of the two groups. Areas of high significance were identified by isocontouring t-statistic maps, calculated as the mean Δdose at each voxel between the outcome groups, scaled by the standard deviation of the Δdose over permutation testing, i.e. the difference due to chance [12], [13].

The registration of each patient CT was visually inspected, focussing on heart and lung placement. Cases with failed registration were removed from the analysis. To account for registration inaccuracies, each deformed dose distribution was blurred by a 3-dimensional Gaussian filter with a width along each axis equivalent to the uncertainty in the deformable registration. This uncertainty was previously estimated by McWilliam et al. by determining the standard deviation of the centre of mass coordinates of deformed heart contours.

The average Δdose and average planned dose within the isosurface defined at 80% of the maximum t-statistic on the significance map relating Δdose to 1 year survival were extracted for each patient. Elastic net penalized Cox regression with equal ridge regression and LASSO penalty terms was then used to select the variables most strongly related to patients’ overall survival. Variables available included: Δdose within the identified region, planned dose within the identified region, age, gender, ECOG-PS, overall stage, T stage, N stage, histology and the gross tumour volume (GTV). The natural logarithm of the GTV was taken to normalise the data. The effect of Δdose in this region on overall survival was then assessed by Cox regression including the clinical factors chosen by the variable selection procedure. The multivariable analysis was repeated in subsets of the data, split according to octiles of the planned region dose to estimate a dose threshold for the observed survival effect. The identified dose threshold for damage was then tested in an independent validation cohort of 1482 NSCLC patients (also treated with 55 Gy in 20 fractions) from the same institution, based upon the planned dose to the region (as shifts were unavailable for this cohort).

## Results

Results of the dose accumulation comparison are shown in Supplementary Materials 2. On average less than 0.1% of the volume of both the whole body and the heart was found to have a dose difference exceeding 1 Gy. Visual inspection showed the largest dose differences occurred far away (superiorly in the left side of the mediastinum and lung) from the regions of interest based on the data mining (located in the right side of the heart). Therefore the assumption of shift-invariance is warranted for this analysis.

Visual assessment of the registrations resulted in 71 registration failures, mostly due to atelectasis in one or both lungs, leaving 475 cases available for image-based data-mining.

**Fig. 1:**
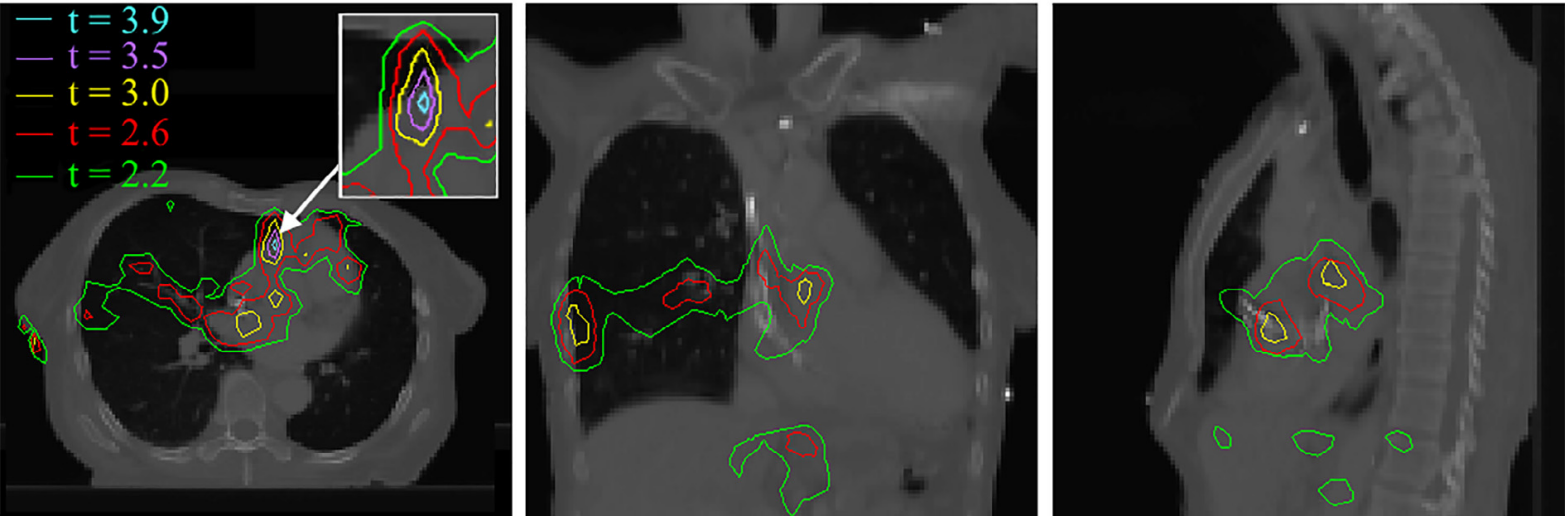
Data mining results of Δdose vs survival at one year. Axial, coronal and sagittal views of the reference patient CT, with the *t*-values overlaid, where the *t*-value provides a measure of the statistical significance of the region over the permutation testing. The most significant region is highlighted by the arrow. Patients who did not survive one year had a significantly higher Δdose in this region.

**Fig. 2:**
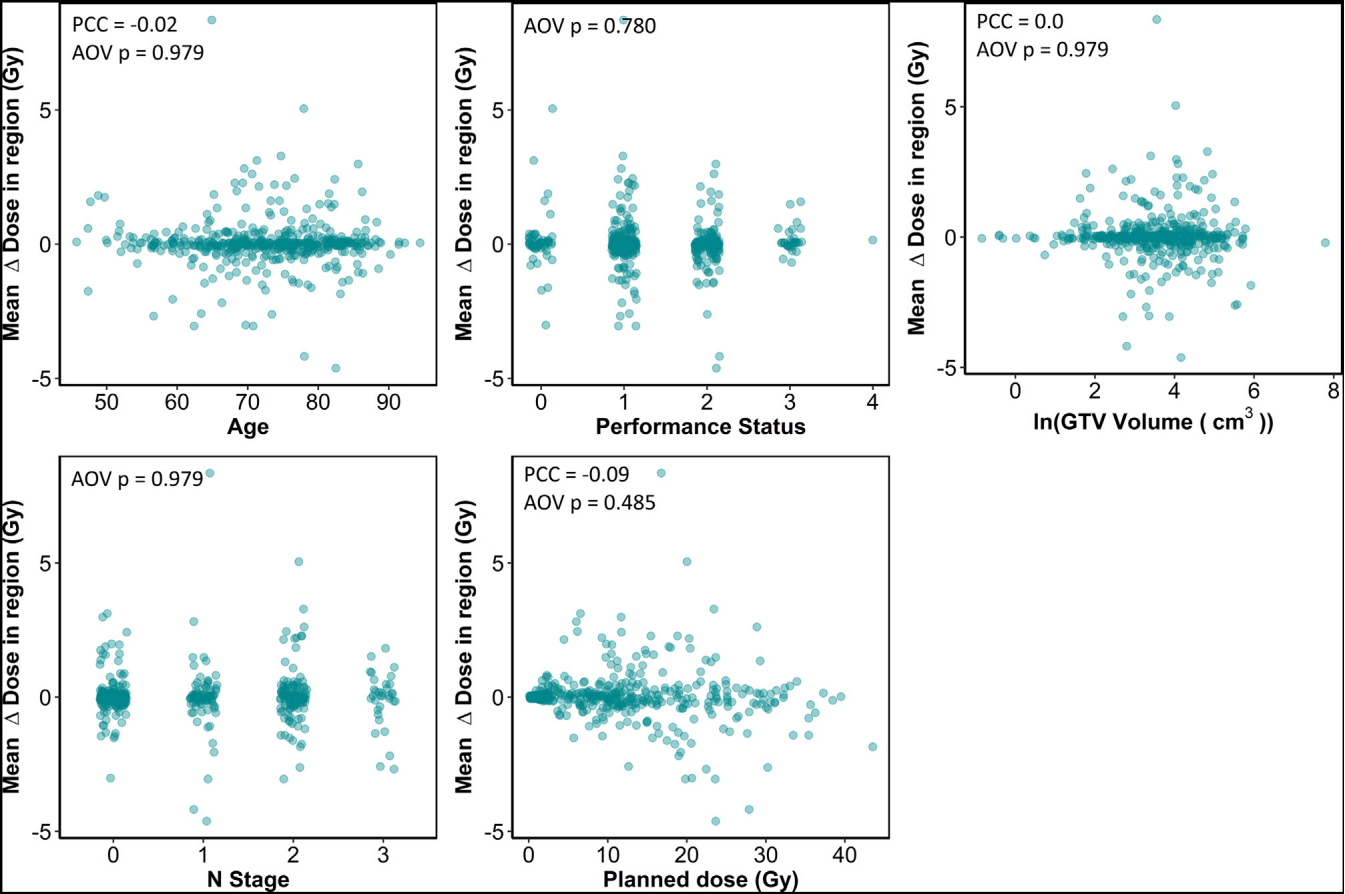
Plots showing the correlation of the mean Δdose within the region of interest shown in Fig. 1 with (a) patient age, (b) performance status, (c) the natural logarithm of GTV volume, (d) N stage and (e) the planned dose in the same region. No correlations are observed, i.e., the dose differences are random.

When Δdose was compared between the patients that did/did not survive 1 year after treatment, a significant difference was observed in a region in the heart base, corresponding, approximately, to the aorta and origin of the coronary arteries (maximum *t*-value = 4.34, *p* = 0.03), as shown in Fig. 1. The mean Δdose in the region defined by 80% of the maximum t-statistic (*t* = 3.5, shown in pink in Fig. 1) ranged between −4.62 Gy and 8.35 Gy over all patients (median 0.02 Gy). No correlations between Δdose in this region and common clinical variables were found, Fig. 2, i.e. the residual setup errors driving this dose difference appear random, yet patients where this random dose difference was higher (on average) in the base of the heart died earlier.

**Fig. 3:**
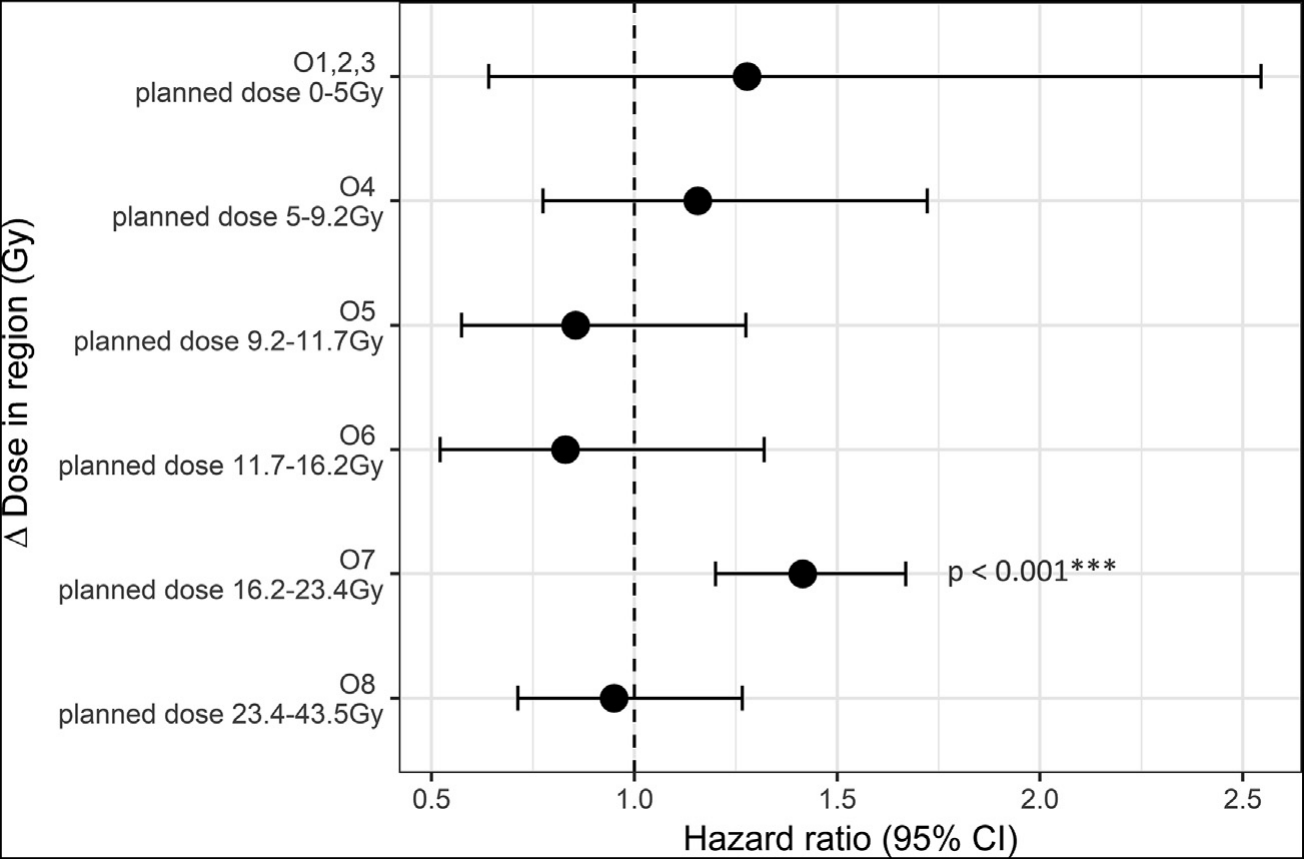
Forest plot showing the hazard ratios and 95% confidence intervals of the mean Δdose in the identified heart region found from multivariate analysis in each planning dose octile. Δdose is only significant in the 7th octile, suggesting a dose threshold for heart damage exists in the range of 16.2–23.4 Gy.

Variable selection found the mean Δdose, planned dose, patient age, ECOG-PS and the natural logarithm of the GTV to be related to overall survival. Table 1 shows the multivariable Cox model results including these variables for the whole cohort. The hazard ratio (HR) for Δdose of 1.221 per Gy demonstrates an increased risk of death with increasing Δdose in the region of interest within the heart (positive difference = increased dose from plan). The resulting octiles, based upon the planned dose in the identified region were: 1st octile 0–1 Gy, 2nd octile 1–2 Gy, 3rd octile 2–5 Gy, 4th octile 5–9.2 Gy, 5th octile 9.2–11.7 Gy, 6th octile 11.7 Gy–16.2 Gy, 7th octile 16.2–23.4 Gy and 8th octile 23.4–43.5 Gy. The first three octiles were combined to obtain a similar range of planned doses as for the other octiles. As shown in Fig. 3, Δdose was only significant in the 7th octile in multivariable analysis, suggesting a steep dose–effect relation for heart damage exists in the identified heart region between 16.2 Gy and 23.4 Gy (although the small size of the sub-cohorts limits significance). For full multivariable results for all octiles see Supplementary Materials 3.

**Table 1:** Multivariable Cox regression hazard ratios (HR) and p-values for the whole cohort using the mean dose difference in the identified region as a continuous variable.

Variable	Hazard Ratio (CI)	*P*-value
Mean Δdose	1.216 (1.085–1.363)	**<0.001**
Ln (GTV)	1.456 (1.318–1.608)	**<0.001**
Age	1.013 (1.002–1.025)	**0.023**

ECOG-PS (0 reference)		
1	1.314 (0.930–1.857)	0.122
2	1.769 (1.234–2.537)	**0.002**
3	1.490 (0.889–2.497)	0.130
4	3.291 (0.450–24.065)	0.241
Planned dose to region	1.013 (1.002–1.025)	**0.024**

**Fig. 4:**
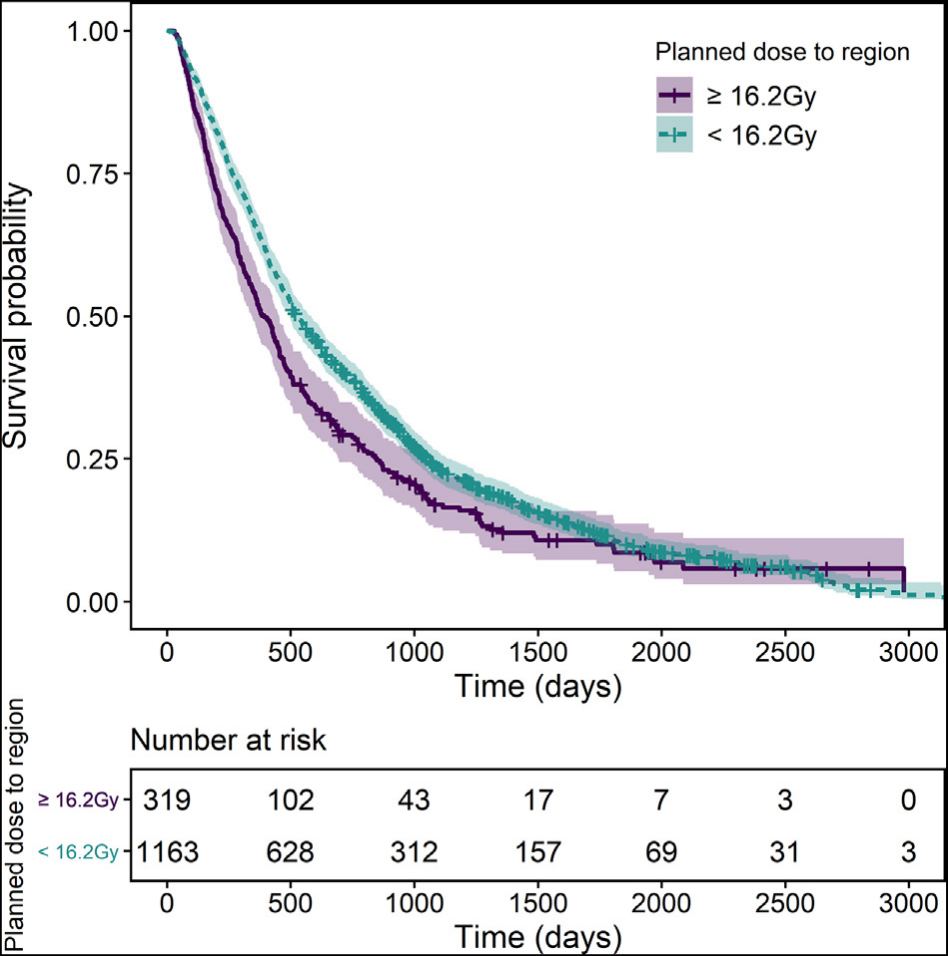
Kaplan–Meier curves showing the difference in survival in the validation cohort between patients who had a planned dose greater than or less than 16.2 Gy to the identified region in the heart. Patients with a higher planned dose in this region had significantly worse outcome.

Fig. 4 shows the corrected Kaplan-Meier plot of overall survival in the validation dataset, with 16.2 Gy taken as a conservative threshold. A significant difference is seen between patients with a region dose above or below 16.2 Gy, with those with a higher heart region dose having worse overall survival (HR_<16.2Gy_ = 0.77, *p* < 0.001). In two subsets of the validation cohort with heart regions doses above or below the 7th octile values (<16.2 Gy or > 23.4 Gy, *n* = 1163 and *n* = 151, respectively), no significant difference in survival was observed when their median dose to the heart region was used as a cut-point (*p* = 0.1 and *p* = 0.4, respectively), providing further confidence in our result.

## Discussion

We identified a region in the base of the heart where differences between planned and delivered dose appears to drive the previously observed survival difference between patients with residual set-up errors which move the heart towards or away from the high dose region [7].

Using a cohort of 475 NSCLC patients, accumulated doses distributions including residual setup errors were estimated and analysed using image-based data-mining. We found that Δdose differs in patients that did and did not survive 1 year, with the most significant region for this effect located within the base of the heart (Fig. 1). We chose to study Δdose instead of accumulated dose, as it is independent of common clinical variables (Fig. 2), and thus provides a cleaner signal, which is not confounded. Δdose was included in multivariable analysis, which revealed it to be significantly predictive of survival, with a hazard ratio of 1.221 per Gy (Table 1). As expected, an increase from the prescribed heart dose results in greater risk of death, with the greatest effect most likely between approximately 16 Gy–24 Gy (when the data is analysed in octiles of the planned region dose). To summarize, the previously observed relation between residual setup errors and survival [7] can be explained by small changes in heart base dose from that planned, incurred as a result of inexact patient setup.

Δdose was significant in multivariable analysis despite the planned dose to the identified region being included, suggesting it is a separate effect that interacts with planned heart dose. When the cohort was split into octiles, Δdose in the heart region was only significant in the 7th octile, with planned region doses between 16.2 and 23.4 Gy. This suggests that a threshold dose exists, above which a steep dose–effect relationship is observed. Below this threshold, changes in dose have little effect. The lack of significance in the 8th octile suggests above 23.4 Gy the dose effect relationship plateaus, where cardiac damage is always incurred. Due to the small cohort sizes and broad range of planned doses in the 8th octile, the analysis should be repeated in a larger cohort to confirm the upper and lower thresholds, and obtain a more accurate estimate of the dose–effect curve.

The volume of evidence on the impact of thoracic radiation on heart dose and survival in lung cancer is significant and growing. The RTOG 0617 phase 3 trial reported worse outcomes in the higher dose arm, which had higher lung and heart doses. Vivekanandan et al. [15] found a significant association between the heart volume receiving 63–69 Gy and survival, with larger volumes associated with higher death rates. The maximum heart dose observed in our study was lower, at 43.5 Gy. This can be explained by the lower prescription dose of 55 Gy in our cohort (Vivikanadan et al. treat with 63–73 Gy) and that we limit our dose assessment to a region of the heart instead of the whole volume. McWilliam et al. [13] identified a region in the base of the heart, and the dose to this region was used to split patients into groups with significantly different overall survival. The cohort in that study was larger but only used planned dose. A first quartile cut point of 8.5 Gy was reported, suggesting a lower threshold for a dose–effect than observed in our study.

Our study has limitations. First, we assumed dose shift invariance, without recalculation of the dose, which may result in inaccuracies, particularly at air-tissue boundaries. For the 13 patients for whom dose accumulation was performed including recalculation, differences were observed only in very small regions. The largest differences (mean range over all patients −2.27 to 2.54 Gy) occurred in small volumes far from the identified regions of interest. The mean dose difference in the heart over all patients was 0.026 Gy, with a standard deviation of 0.058 Gy*.* Neither of our applied accumulation methods take anatomical changes through-out the course of treatment into account. We expect anatomical changes to be randomly distributed throughout the whole cohort, and as our cohort was split using a random variable (Fig. 2), to thus be randomly distributed in the two arms. However, until validation can be performed in cohorts with additional imaging, e.g. after treatment delivery as well as before, for every treatment fraction, then the obtained Δdose should be taken only as an indication of the change in dose and interpreted with care.

Second, the method to estimate the residual setup errors is crude and will likely underestimate the residual shifts [1]. Image-guidance was performed using 3D-CBCTs, which will not take into account respiratory motion. We thus assumed a static heart position when determining the residual errors, using the centre of mass of the heart contour as a representative point. Several studies report heart motion due to the cardiac cycle and respiration [16], [17]. In addition, we assume the heart position is stable relative to the bony anatomy. It is therefore important that our work is validated in patient cohorts where the heart shift can be estimated more accurately, i.e. based on daily imaging data using large field 4D-CBCT with the heart inside the field-of-view. This would also allow for more accurate dose accumulation to investigate the potential role of anatomical changes. However, for now these uncertainties are ignored. However, these effects will be independent of the residual setup errors and therefore of Δdose, so our analysis remains valid.

Finally, we observed dose differences inside and outside the heart (Fig. 1). We assume the locations outside the heart are highlighted due to implicit correlations, as an increased dose in one location will affect dose elsewhere along commonly used beamlines.

The region identified in this analysis is in a similar location to that observed by McWilliam et al*.* however, the shape is different. The previous study identified a region that extended from the heart in the anterior-posterior direction, while our results show more lateral spread (Fig. 1). This is likely because the dose differences have different drivers: planned dose is heavily confounded, and primarily affected by tumour size and location, while Δdose is driven by the independent setup errors. It is possiblethat the intersection of the regions contains the actual anatomy that is responsible for early mortality. This region includes the origins of the coronary arteries and the conduction system (e.g. sino-atrial node). Prospective measurements of heart function after radiotherapy are ongoing to identify the underlying physiology of heart toxicity.

In this study we localize a region in the base of the heart where changes to the planned dose resulting from residual errors following image-guidance correlate with survival, with a steep dose–effect relation in the range of 16–23 Gy – which was confirmed in an independent validation set. The shape of the dose–response curve is not yet clear, but our method can be applied to larger cohorts to establish a more precise threshold dose. Our results suggest that stricter imaging protocols should be used, to ensure the heart dose is not increased unnecessarily, and stricter planning dose constraints should be imposed, as parts of the heart appear to be sensitive to radiation dose. Important unanswered questions are to exactly define the regions of the heart to avoid during the radiotherapy planning process and the appropriate heart dose constraint to reduce early mortality.

## Conflict of interest

None.
