# Supplementary material for: Impact of small residual setup errors after image guidance on heart dose and survival in non-small cell lung cancer treated with curative-intent radiotherapy
Source: Radiother Oncol. 2020 Nov;152:177–82. doi: 10.1016/j.radonc.2020.04.008 (PMC7707351; doi:10.1016/j.radonc.2020.04.008)
Supplement: Supplementary Data 1 [file mmc1.docx]

# Supplementary Table 1: Patient cohort

| Variable | NSCLC cohort (n = 475) |
| --- | --- |
| Mean Age (range) | 73 (45 – 94) |
| Gender |  |
| Male | 232 (48.84%) |
| Female | 243 (51.16%) |
| ECOG-PS |  |
| 0 | 56 (11.79%) |
| 1 | 199 (41.89%) |
| 2 | 148 (31.16%) |
| 3 | 31 (6.53%) |
| 4 | 1 (0.21%) |
| Missing | 40 (8.42%) |
| Comorbidities |  |
| 0 | 33 (6.95%) |
| 1 | 62 (13.05%) |
| 2 | 66 (13.89%) |
| 3 | 49 (10.32%) |
| Missing | 265 (55.79%) |
| Stage |  |
| I | 10 (2.11%) |
| II | 88 (18.53%) |
| III | 295 (62.11%) |
| IV | 23 (4.84%) |
| Missing | 59 (12.42%) |
| T stage |  |
| 1 | 57 (12.00%) |
| 2 | 187 (39.37%) |
| 3 | 129 (27.16%) |
| 4 | 74 (15.58) |
| Missing | 28 (5.89%) |
| N stage |  |
| 0 | 206 (43.37%) |
| 1 | 84 (17.68%) |
| 2 | 124 (26.11%) |
| 3 | 34 (7.16%) |
| Missing | (5.68%) |
|  |  |
| Histology |  |
| Adenocarcinoma | 188 |
| Squamous Cell Carcinoma | 186 |
| Other | 11 |
| Not Otherwise Specified | 90 |
| Mean GTV cm^3^ | 63 |
| Missing | 56 (11.79%) |

# Supplementary Material 2: dose accumulation

To validate our simple ‘shift and sum’ dose accumulation method, we compared the dose at each point to the dose distribution recalculated following daily isocentre shifts for 13 patients. These comparisons were first performed at each point within the body contour minus a 2cm rind for each patient – selected to exclude the surface region from analysis, as it will inherently suffer from dose differences due to the simple shifting method. We repeated the analysis for pixels within the heart contours. Mean, median, maximum, minimum and standard deviation of the dose difference were reported, along with the percentage of the volume in which the accumulated doses differed by more than 1Gy, for each structure, as shown below.

It can be seen that in the majority of the volume there is no appreciable difference between the two methods, with on average less than 0.1% of the volume having a dose difference exceeding 1Gy. The use of this method, and therefore the assumption of shift-invariance, is therefore justified for this analysis.

| Body – 2cm | | | | | | | Heart | | | | | |
| --- | --- | --- | --- | --- | --- | --- | --- | --- | --- | --- | --- | --- |
| Patient | Max. Diff. (Gy) | Min. Diff. (Gy) | Median Diff. (Gy) | Mean Diff. (Gy) | % Volume with < ‑1 Gy Diff. | % Volume with > 1 Gy Diff. | Max. Diff. (Gy) | Min. Diff. (Gy) | Median Diff. (Gy) | Mean Diff. (Gy) | % Volume with < ‑1 Gy Diff. | % Volume with > 1 Gy Diff. |
| 1 | 2.623 | -3.156 | -0.006 | -0.027 | 0.236 | 0.072 | 1.675 | -1.347 | 0.003 | -0.037 | 0.000 | 0.010 |
| 2 | 0.967 | -0.650 | -0.005 | -0.001 | 0.000 | 0.000 | 0.500 | -0.289 | -0.008 | -0.008 | 0.000 | 0.000 |
| 3 | 3.820 | -1.899 | -0.005 | 0.003 | 0.032 | 0.126 | 0.762 | -0.932 | 0.002 | -0.002 | 0.000 | 0.000 |
| 4 | 2.001 | -1.028 | -0.004 | 0.000 | 0.000 | 0.036 | 0.765 | -0.577 | -0.018 | -0.018 | 0.000 | 0.000 |
| 5 | 1.736 | -1.746 | -0.001 | 0.030 | 0.036 | 0.043 | 0.527 | -0.506 | 0.055 | 0.068 | 0.000 | 0.000 |
| 6 | 3.161 | -1.393 | -0.003 | 0.004 | 0.015 | 0.064 | 2.669 | -0.984 | 0.025 | 0.040 | 0.000 | 0.157 |
| 7 | 2.434 | -2.732 | -0.004 | 0.000 | 0.167 | 0.113 | 1.231 | -1.204 | -0.002 | 0.011 | 0.001 | 0.000 |
| 8 | 1.747 | -1.589 | -0.002 | 0.026 | 0.038 | 0.038 | 0.791 | -0.661 | 0.049 | 0.051 | 0.000 | 0.000 |
| 9 | 4.202 | -3.029 | -0.005 | -0.013 | 0.250 | 0.215 | 4.048 | -2.473 | -0.003 | -0.027 | 0.215 | 0.445 |
| 10 | 2.918 | -2.345 | -0.006 | -0.001 | 0.113 | 0.049 | 1.969 | -1.113 | 0.097 | 0.105 | 0.000 | 0.008 |
| 11 | 2.330 | -3.128 | -0.004 | 0.011 | 0.035 | 0.013 | 0.681 | -0.709 | 0.004 | 0.000 | 0.000 | 0.000 |
| 12 | 3.246 | -4.953 | -0.007 | 0.010 | 0.289 | 0.229 | 0.979 | -0.985 | -0.011 | -0.010 | 0.023 | 0.000 |
| 13 | 1.892 | -1.888 | -0.002 | 0.034 | 0.021 | 0.207 | 1.826 | -0.583 | 0.110 | 0.161 | 0.000 | 0.552 |
| Mean (SD) | 2.544 (0.911) | -2.272 (1.136) | -0.004 (0.002) | 0.006 (0.017) | 0.095 (0.105) | 0.093 (0.079) | 1.417 (1.028) | -0.951 (0.549) | 0.023 (0.042) | 0.026 (0.058) | 0.018 (0.059) | 0.090 (0.188) |

Results of the dose accumulation comparison. Tabulated values are extracted from the dose difference map calculated as the dose distribution accumulated under the assumption of shift invariance (i.e. by simply shifting the daily dose distributions) subtracted from the dose distributions recalculated following daily isocentre shifts. Values are reported within the body contour minus a 2cm rind (left) and heart contours (right).

# Supplementary Materials 3:

| Dataset | Variable | Hazard Ratio (CI) | P-value |
| --- | --- | --- | --- |
| Combined 1st, 2nd | Mean Δdose | 1.430 (0.737–2.775) | 0.290 |
| and 3rd octiles | Ln(GTV) | 1.514 (1.261–1.818) | **<0.001** |
| (planned dose: 0-5Gy) | Age | 1.000 (0.981–1.019) | 0.970 |
| (N=178) | ECOG-PS (0 reference) |  |  |
|  | 1 | 1.733 (0.980–3.067) | 0.059 |
|  | 2 | 1.916 (1.070–3.429) | **0.029** |
|  | 3 | 1.587 (0.702–3.588) | 0.267 |
|  | 4 | - | - |
|  | Planned dose to region | 1.047 (0.889–1.233) | 0.581 |
|  |  |  |  |
| 4th octile | Mean Δdose | 1.097 (0.727–1.656) | 0.657 |
| (planned dose: 5-9.2Gy) | Ln(GTV) | 1.380 (1.020–1.868) | **0.037** |
| (N=60) | Age | 1.016 (0.979–1.055) | 0.395 |
|  | ECOG-PS (0 reference) |  |  |
|  | 1 | 0.525 (0.125–2.212) | 0.380 |
|  | 2 | 1.432 (0.318–6.444) | 0.640 |
|  | 3 | 0.780 (0.130–4.683) | 0.786 |
|  | 4 | - | - |
|  | Planned dose to region | 0.846 (0.653–1.097) | 0.206 |
|  |  |  |  |
| 5th octile | Mean Δdose | 0.908 (0.620–1.330) | 0.620 |
| (planned dose: 9.2-11.7Gy) | Ln(GTV) | 2.672 (1.705–4.188) | **<0.001** |
| (N=59) | Age | 1.083 (1.030–1.139) | **0.002** |
|  | ECOG-PS (0 reference) |  |  |
|  | 1 | 1.504 (0.398–5.681) | 0.548 |
|  | 2 | 1.214 (0.316–4.669) | 0.778 |
|  | 3 | 7.256 (0.970–54.313) | 0.054 |
|  | 4 | 4.452 (0.408–48.612) | 0.221 |
|  | Planned dose to region | 0.867 (0.590–1.274) | 0.468 |
|  |  |  |  |
| 6th octile | Mean Δdose | 0.919 (0.583–1.448) | 0.715 |
| (planned dose:11.7-16.2Gy) | Ln(GTV) | 1.857 (1.274–2.706) | **0.001** |
| (N=60) | Age | 0.981 (0.949–1.013) | 0.236 |
|  | ECOG-PS (0 reference) |  |  |
|  | 1 | 2.556 (0.811–8.063) | 0.109 |
|  | 2 | 3.713 (1.143–12.061) | **0.029** |
|  | 3 | 2.244 (0.490–10.273) | 0.298 |
|  | 4 | - | - |
|  | Planned dose to region | 0.831 (0.649–1.064) | 0.143 |
|  |  |  |  |
| 7th octile | Mean Δdose | 1.337 (1.155–1.547) | **<0.001** |
| (planned dose: 16.2-23.4Gy) | Ln(GTV) | 1.228 (0.925–1.632) | 0.156 |
| (N=58) | Age | 1.023 (0.987–1.061) | 0.211 |
|  | ECOG-PS (0 reference) |  |  |
|  | 1 | 1.898 (0.747–4.828) | 0.178 |
|  | 2 | 2.465 (0.846–7.182) | 0.098 |
|  | 3 | 2.750 (0.522–14.500) | 0.233 |
|  | 4 | - | - |
|  | Planned dose to region | 1.099 (0.932–1.295) | 0.262 |
|  |  |  |  |
| 8th octile | Mean Δdose | 1.014 (0.790–1.302) | 0.913 |
| (planned dose: 23.4-43.5Gy) | Ln(GTV) | 1.133 (0.834–1.540) | 0.425 |
| (N=60) | Age | 1.062 (1.021–1.105) | **0.003** |
|  | ECOG-PS (0 reference) |  |  |
|  | 1 | 1.099 (0.423–2.853) | 0.847 |
|  | 2 | 0.880 (0.324–2.391) | 0.802 |
|  | 3 | 5.286 (0.526–53.169) | 0.157 |
|  | 4 | - |  |
|  | Planned dose to region | 1.048 (0.977–1.123) | 0.190 |

*Multivariable Cox regression hazard ratios (HR) and p-values for subsets of the data, created by dividing the cohort into equal octiles based upon the planned dose to the identified region (1^st^ octile 0 – 1Gy, 2^nd^ octile 1 – 2Gy, 3^rd^ octile 2 – 5Gy, 4^th^ octile 5 – 9.2Gy, 5^th^ octile 9.2 – 11.7Gy, 6^th^ octile 11.7Gy – 16.2Gy, 7^th^ octile 16.2 – 23.4Gy and 8^th^ octile 23.4 – 43.5Gy).*
